# Analysis of filaggrin 2 gene polymorphisms in patients with atopic dermatitis^☆^^☆☆^

**DOI:** 10.1016/j.abd.2019.07.002

**Published:** 2020-01-25

**Authors:** Amanda Hertz, Luna Azulay-Abulafia, Adriana Paulino do Nascimento, Cintya Yumi Ohara, Fabio Chigres Kuschnir, Luís Cristovão Porto

**Affiliations:** a Pediatric Dermatology Clinic, Instituto de Dermatologia Prof. Rubem David Azulay, Rio de Janeiro, RJ, Brazil; b Department of Dermatology, Instituto de Dermatologia Prof. Rubem David Azulay, Universidade do Estado do Rio de Janeiro, Rio de Janeiro, RJ, Brazil; c Histocompatibility and Cryopreservation Laboratory, Universidade do Estado do Rio de Janeiro, Rio de Janeiro, RJ, Brazil; d Department of Allergy and Immunopathology, Universidade do Estado do Rio de Janeiro, Rio de Janeiro, RJ, Brazil; e Department of Pediatrics, Faculdade de Ciências Médicas, Universidade do Estado do Rio de Janeiro, Rio de Janeiro, RJ, Brazil; f Department of Allergy, Universidade do Estado do Rio de Janeiro, Rio de Janeiro, RJ, Brazil

**Keywords:** Allergy, Dermatitis, atopic, Immunology, Polymorphism, genetic

## Abstract

**Background:**

Polymorphisms of the filaggrin 2 gene (rs 12568784 and rs 16899374) are associated with persistent atopic dermatitis in African American patients. Filaggrin 2 is a protein with a function similar to filaggrin and also encoded in the epidermal differentiation complex on chromosome 1q21.

**Objective:**

To evaluate the polymorphisms in the filaggrin 2 gene (rs 12568784 and rs 16899374) in children and adults with atopic dermatitis and to verify the association of these with the severity of the clinical picture, presence of other allergic diseases, and socio-demographic factors.

**Method:**

The study was carried out with patients and control group. Questionnaires were used to evaluate ethnicity, sex, age, family history, scoring, atopic dermatitis (SCORAD), among other parameters. Genotyping of the filaggrin 2 gene was performed by real-time polymerase chain reaction.

**Results:**

Forty-eight patients and 83 controls were evaluated. No correlation was found between the variables studied in patients with atopic dermatitis and polymorphisms, no significant difference between the prevalence of polymorphisms in the patients and in the control group *p* > 0.05.

**Study limits:**

The exclusive use of self-reported ethnicity information and the sample size.

**Results:**

The results of this work can be an incentive for the study of the polymorphisms in atopic dermaititis, considering the characteristic of the Brazilian multi ethnic population.

**Conclusion:**

This is an unpublished work in Brazil and the first study in the world to have a control group to evaluate alterations in the gene of filaggrin 2.

## Introduction

Atopic dermatitis (AD) is a frequent inflammatory skin disease that affects approximately 20% of children, compromising the quality of life of the patient and the family. Mutations in the gene encoding the filaginous epidermal protein have been shown to be an important factor in the development of this disease.^1^ Filaggrin is also associated with the persistence and coexistence of other allergic diseases.^2^

The filaggrin (FILAGGRIN: FILamentAGGRegatingproteIN) is a protein found in corneocytes responsible for aggregating keratin in the formation of stratum corneum.^3^ It is produced from a precursor, pro-filaggrin, which is located in the granular layer of the epidermis in the granules of keratohyalin. Pro-filaggrin is subsequently dephosphorylated, resulting in the formation of filaggrin monomers having keratin filament aggregation properties. Filaggrin degradation generates Natural Moisturizing Factors (NMFs), which include free amino acids, Urocanic Acid (UCA), and Carboxylic Pyrrolidonic Acid (PCA). NMFs promote water uptake and entrapment, and maintenance of acidic pH of the skin, essential for the process of hydration and maintenance of the skin barrier.^4^

The gene encoding filaggrin is located on chromosome 1q21, in a region called epidermal differentiation complex.^3^ According to the literature, 27.5% of Caucasian Americans, 48% of Europeans, 31.4% of Chinese and 20% of Japanese with AD present mutations in the filaggrin gene.^5^

Currently, 47 mutations with loss of function have already been identified in the gene encoding filaggrin in patients with AD. Profiles other than mutations were identified in patients from Europe and Asia.^6^ Among the mutations described, the most commonly found in Caucasians are R501X and 2282del4 (comprising 50% of the total), in addition to R2447X and S3247X.^7^ In the Japanese population, the main mutations were 3321delA and S2554X.^8^ These mutations seem to vary according to ethnic groups, for example, in the African-American population studies have shown a much lower prevalence than in Caucasians.^9^ Gao et al. found that R501X was found in 3.2% of 187 African-American patients with AD and no association with 2282 of the 4 patients.^10^ Margolis et al. evaluated the genotype of 370 African Americans with AD and found mutations in only, 1% of patients (R501X, 3.2%; 2282del4, 0.5%; S3247X, 3% and R2447X, 1.4%).^7^, ^8^

Filaggrin 2 is a protein whose function is similar to filaggrin and also encoded in the region called epidermal differentiation complex, according to Makino et al. the expression of filaggrin 2 protein is decreased in patients with AD.^11^ There is an association between polymorphisms in the filaggrin 2 gene (rs 12568784 and rs 1689937411) and persistent AD in African American patients with atopic dermatitis.^12^

The aim of this study was to evaluate the polymorphisms in the filaggrin 2 gene (rs 12568784 and rs 16899374) in children and adults with atopic dermatitis and to verify the association of these with the severity of the clinical picture, presence of other allergic diseases and socio-demographic factors.

## Methods

A cross-sectional study involving patients of both sexes, aged between 1 and 27 years, diagnosed with atopic dermatitis, according to United Kingdom Working Party (UK) criteria,^13^ recruited from outpatient clinics for atopic dermatitis. Patients with other chronic diseases of the skin (psoriasis, among others) or systemic diseases that affect the skin, such as primary immunodeficiencies, systemic lupus erythematosus were excluded.

According to the order of arrival in the clinic, the patients answered a questionnaire with the following variables: gender, age, ethnicity, onset of AD, family history of allergic diseases and presence of asthma, food allergy and rhinitis. The ISAAC questionnaire, validated for our language^14^, ^15^ was used to evaluate the presence of asthma and rhinitis. In addition, the severity of AD was analyzed by SCORAD (Scoring Atopic Dermatitis).^14^, ^15^ The severity levels of AD were defined as light: SCORAD up to 25 points; moderate between 25–50 points; and serious, above 50 points.^16^

After signing of consent terms, venous blood samples were collected in tubes containing EDTA. In order to increase the reliability of the study and because of the ethical difficulty of creating a pediatric control group with blood collection, control samples from the same population group from a bone marrow bank were chosen, which were matched by sex and ethnicity.

To determine the genetic variation of filaggrin 2, the genomic DNA of the individuals participating in the study was purified using the QIAamp DNA Blood Mini Kit (Qiagen, Valencia, CA) according to the manufacturer's instructions. The principle of this kit is based on the extraction of genomic DNA from nucleated blood cells and subsequent binding of the DNA to a glass fiber matrix contained in a MicroSpin column. Briefly, after digestion of the cell membranes by proteinase K, the nucleic material was transferred to a silica membrane where it was subjected to several washes with alcoholic solutions. The elution was done by the addition of ultrapure water. The genomic DNA was quantified by measuring the absorbance at the optical density of 260 nm (OD260) using the NanoDrop Lite spectrophotometer (ThermoScientific). The final preparation was stored at −20 °C.

Genotyping of the filaggrin 2 gene (rs16833974 and rs12568784) was performed by real-time PCR using commercially available TaqMan® (ABI assay; AppliedBiosystems) probe labeled with the VIC and FAM fluorophores for each allele, C_34256909_10 for rs16833974 and C_11261511 to rs12568784. Briefly, the reaction was performed in a final volume of 10 μL containing 40 ng of genomic DNA, Taqman SNP Genotyping Assays 40X and TaqmanGenotyping Master Mix according to the manufacturer's instructions. Initially the samples were subjected to a pre-PCR reading step at 60 °C for 30 s, followed by the denaturation step at 95 °C for 10 min. Then the amplification stage consisted of 40 cycles of 95 °C for 15 s and 60 °C for 1 min, for annealing of the primer and extension of the fragments. After amplification of the DNA fragments, the samples were submitted to the final post-PCR reading step at 60 °C for 30 s. The PCR reaction was performed with the aid of the ABI StepOnePlus Real-Time PCR System (AppliedBiosystems, Foster City, CA). The results of the allelic discrimination analysis were plotted on a scatter plot contrasting the fluorescence of each probe. In order to increase the reliability of the study in relation to mutation analysis, control samples from a bone marrow bank were analyzed.

### Statistical analysis

A sample of 48 patients with a larger control group (1.7 times) of 83 patients was used for a better statistical analysis. The frequency and relative frequency statistics were used to present the variables in descriptive tables, in order to understand the profile of the sample and also of the controls. In order to verify possible associations, the Chi-square test, or Fisher's exact test, was used to identify evidence that shows the significance of certain variables in relation to the genetic mutation studied and SCORAD (Scoring atopic dermatitis) level.

The significance level of 0.05, *p*-value <0.05 was used to evaluate the associations between the dependent variable (mutations in the filaggrin 2 gene) and the other variables of interest in the study. The programs Microsoft Excel 2010 and Software R, version 3.3.1 (R Core Team 2015, Vienna, Austria) were employed for organizing, creating tables and statistical analysis of data. The study followed the principles of the Helsinki Declaration and was approved by the ethics committee under opinion n°36934514.9.0000.5259.

## Results

From November 2015 to February 2016 data were collected from 48 patients, 54% male. The control group consisted of 83 samples. The total polymorphism prevalence rs 16833974 in the group of patients was 16.7% (14.6% heterozygote and 2.1% homozygous) and 15.7% in the control group (15.7% heterozygous) and the total prevalence of rs polymorphism rs 12568784 were: 43.7% of the patients (35.4% heterozygous and 8.3% homozygous) and control: 46.9% (37.3% heterozygous and 9.6% homozygous). The main characteristics of the sample and the control group are described in table 1.

**Table 1:** Distribution of socio-demographic data of patients and controls

Sociodemographics variables	Patients			Control		
	*n*	%		*n*	%	
*Sex*						
F	22	46%		33	40%	0.58
M	26	54%		50	60%	

*Ethnicity*						
White	17	35%		29	35%	
Brown	12	25%		26	31%	0.51
African american	18	38%		28	34%	
Unidentified	1	2%		0	0%	

*Age*						
Infant (0–2 years)	4	8%	21–30 years	55	66%	
Children (3–9 years)	28	58%	31–45 years	21	25%	
Adolescent (10–19 years)	13	27%	>45 years	6	7%	
Adult (20 years or more)	3	6%	Unidentified	1	1%	

The genetic polymorphism distribution of rs 16833974 and rs 12568784 according to the socio demographic variables of the sample and the control group are respectively shown in table 2.

**Table 2:** Distribution of the rs16833974 and rs12568784 polymorphism according to the sociodemographic variables of the patients and control group

Sociodemographics variables	Polymorphism rs16833974									
	Patients					Control				
	Present		Absent		*p*-Value	Present		Absent		*p*-Value
	*n*	%	*n*	%		*n*	%	*n*	%	
*Sex*										
F	4	8%	18	38%	1	5	6%	28	34%	0.14
M	4	8%	22	46%		8	10%	42	51%	

*Ethnicity*										
White	1	2%	16	33%	0.08	1	1%	28	34%	0.03
Brown	1	2%	11	23%		4	5%	22	27%	
African American	6	13%	12	25%		8	10%	20	24%	
Unidentified	0	0%	1	2%		0	0%	0	0%	

Sociodemographics variables	Polymorphism rs12568784									
	Patients					Control				
	Present		Absent		*p*-Value	Present		Absent		*p*-Value
	*n*	%	*n*	%		*n*	%	*n*	%	
*Sex*										
F	11	23%	11	23%	0.56	19	23%	14	17%	0.17
M	10	21%	16	33%		20	24%	30	36%	

*Ethnicity*										
White	7	15%	10	21%	0.38	10	12%	19	23%	0.51
Brown	3	6%	9	19%		17	20%	9	11%	
African American	10	21%	8	17%		12	14%	16	19%	
Unidentified	1	2%	0	0%		0	0%	0	0%	

The main variables studied were: gender, ethnicity, SCORAD, family history of atopy and associated allergic diseases. As for polymorphism rs 16833974, statistically significant differences were found regarding the ethnicity variable, and the darker the skin color the greater the chance of having the polymorphism, *p*-value = 0.031 (Table 3).

**Table 3:** Variables studied in the patient's group and polymorphism rs16833974

Variables	Polymorphism rs 16833974				Total		*p*-Value
	Present		Absent		*n*	%	
	*n*	%	*n*	%			
*Sex*							
F	4	50%	18	45%	22	46%	1
M	4	50%	22	55%	26	54%	
Total	8	100%	40	100%	48	100%	

*Scorad*							
Mild	6	75%	13	33%	19	40%	0.026
Moderate	2	25%	15	38%	17	35%	
Severe	0	0%	10	25%	10	21%	
Unidentified	0	0%	2	5%	2	4%	
Total	8	100%	40	100%	48	100%	

*HF Atopia*							
Yes	8	100%	33	83%	41	85%	0.56
No	0	0%	5	13%	5	10%	
Unidentified	0	0%	2	5%	2	4%	
Total	8	100%	40	100%	48	100%	

*Mother*							
Yes	5	63%	18	45%	23	48%	0.7
No	3	38%	17	43%	20	42%	
Unidentified	0	0%	5	13%	5	10%	
Total	8	100%	40	100%	48	100%	

*Father*							
Yes	6	75%	16	40%	22	46%	0.24
No	2	25%	19	48%	21	44%	
Unidentified	0	0%	5	13%	5	10%	
Total	8	100%	40	100%	48	100%	

*Ethnicity*							
White	1	13%	16	40%	17	35%	0.031
Brown	1	13%	11	28%	12	25%	
African American	6	75%	12	30%	18	38%	
Unidentified	0	0%	1	3%	1	2%	
Total	8	100%	40	100%	48	100%	

*Asthma*							
Yes	4	50%	18	45%	22	46%	0.66
No	2	25%	19	48%	21	44%	
Unidentified	2	25%	3	8%	5	10%	
Total	8	100%	40	100%	48	100%	

*Food allergy*							
Yes	1	13%	15	38%	16	33%	0.37
No	5	63%	22	55%	27	56%	

As for polymorphism rs12568784, the following variables were also studied: sex, ethnicity, SCORAD, family history of atopy and associated allergic diseases (food allergy, asthma and rhinitis) and no statistical significance was found among the variables except for the parent variable. The test had *p*-value = 0.032, which was lower than the significance level of 0.05 (5%). Given this, it is possible to affirm the existence of statistical evidences that prove some causal relation between polymorphism and Atopy (Father). According to the data in the table we can see a reduction in polymorphism rs12568784 in the presence of Atopia (Pai) (Table 4).

**Table 4:** Variables studied in the patient's group and polymorphism rs1256878

Variables	Polymorphism rs12568784				Total		*p*-Value
	Present		Absent		*n*	%	
	*n*	%	*n*	%			
*Sex*							
F	11	52%	11	41%	22	46%	0.56
M	10	48%	16	59%	26	54%	
Total	21	100%	27	100%	48	100%	

*Scorad*							
Mild	9	43%	10	37%	19	40%	0.91
Moderate	5	24%	12	44%	17	35%	
Severe	5	24%	5	19%	10	21%	
Unidentified	2	10%	0	0%	2	4%	
Total	21	100%	27	100%	48	100%	

*Family history atopia*							
Yes	20	95%	21	78%	41	85%	0.059
No	0	0%	5	19%	5	10%	
Unidentified	1	5%	1	4%	2	4%	
Total	21	100%	27	100%	48	100%	

*Mother*							
Yes	11	52%	12	44%	23	48%	1
No	9	43%	11	41%	20	42%	
Unidentified	1	5%	4	15%	5	10%	
Total	21	100%	27	100%	48	100%	

*Father*							
Yes	14	67%	8	30%	22	46%	0.032
No	6	29%	15	56%	21	44%	
Unidentified	1	5%	4	15%	5	10%	
Total	21	100%	27	100%	48	100%	

*Ethnicity*							
White	7	33%	10	37%	17	35%	0.38
Brown	3	14%	9	33%	12	25%	
African American	10	48%	8	30%	18	38%	
Unidentified	1	5%	0	0%	1	2%	
Total	21	100%	27	100%	48	100%	

*Asthma*							
Yes	8	38%	14	52%	22	46%	0.54
No	10	48%	11	41%	21	44%	
Unidentified	3	14%	2	7%	5	10%	
Total	21	100%	27	100%	48	100%	

*Food allergy*							
Yes	6	29%	10	37%	16	33%	0.75
No	12	57%	15	56%	27	56%	
Unidentified	3	14%	2	7%	5	10%	
Total	21	100%	27	100%	48	100%	

A total of 46 crosses of interest were performed in order to verify statistical significance between polymorphism and family history, sex, ethnicity, disease severity, atopy (asthma, rhinitis, familial allergy) and comparison with control of mutation frequency, ethnicity and sex. We sought to perform evaluation separately from heterozygous and homozygous polymorphisms, but no association was found.

## Discussion

The relationship between polymorphisms of the filaggrin 2 gene and AD has recently been reported.^11^ According to the literature, filaggrin 2 polymorphism may influence the severity of the disease.^11^ In this study, we sought to analyze some points of this association in a broad way, through the exploratory analysis and correlation of clinical and demographic data.

It is important to consider that the control group used in the study was of convenience, with samples of bone marrow bank, due to the difficulty of organizing a control group of children. A gender and ethnicity pairing was performed between the groups. This is the first work that uses controls to evaluate polymorphisms of filaggrin 2 and DA. No statistical differences were found between the prevalence of polymorphisms in the group of patients with AD and in the control group. In addition, there was no difference in severity in patients with polymorphism.

Likewise, when we analyzed the correlations between age at onset of AD and polymorphisms, there were no statistical differences. As described by Margolis et al., no association of polymorphism with other atopic-valued diseases (asthma, rhinitis, food allergy) was also found.^12^

Family history is an important risk factor for the development of AD. Approximately 70% of patients with AD present a family history of atopic diseases. The probability of developing AD is about two to three times greater in children with one atopic parent, and increases to three to five times if both parents are atopic.^17^ In general, the maternal history of AD is more predictive for the development of AD in relation to paternal atopy.^18^ In our study, 85% of the patients presented positive family history for atopy, corroborating the literature.

In general, no statistical differences were observed between family history of atopy and filaggrin polymorphisms 2, except for the frequency of the polymorphism mutation rs 12568784 in the presence of paternal atopy. This data can only refer to a statistical finding, due to the small sample number and possibly without biological plausibility. More studies are needed to elucidate this finding.

According to Margolis et al. (2014), filaggrin gene polymorphisms 2, rs 12568784 and rs 16833974, are associated with the persistence of AD in African Americans and are absent or are rarely found in individuals of European origin.^12^ In the study population, there was statistical significance among the patients with autodeclared black AD and the polymorphisms rs 16833974.

The Brazilian population has a high degree of miscegenation and follows different ethnic patterns when compared to population groups from other countries and continents such as China, the United States, Japan and Europe, where there are already studies with the prevalence of filaggrin.^5^ Therefore, it is difficult to establish a classification by ethnicities in our country. The exclusive use of information of the self-declared ethnic group is not the best method for ethnic classification.^19^

The genomes of most Brazilians are miscegenated and genetic markers are capable of providing valuable new insights into the current structure of the Brazilian population.^19^

Future prospects of ancestral analysis of blood samples from our study may allow a better understanding of the association between filaggrin 2 and DA, since the prevalence of filaggrin gene mutations show ethnic differences in both the general population and individuals with DA.^20^

The small sample size may have limited the significance of our results and perhaps a larger sample and/or ancestral study may contribute to the clarification between individuals with AD and filaggrin polymorphisms 2. The performance of the proteical study of filaggrin 2, since structural differences may be due to post-transcriptional controls, would also contribute to a better evaluation of filaggrin 2.

To date, there are no studies of filaggrin 2 and DA gene polymorphisms in Latin America, reinforcing the relevance of our study and the novelty of this initiative in Brazil. In addition, this is the first worldwide study using a control group to evaluate changes in the filaggrin gene 2.

## Conclusion

Very little is known about the profile of filagrins 2 polymorphisms associated with AD in the highly mixed Brazilian population. The results of this work can be a considerable incentive to research on polymorphisms and AD in the country, collaborating to a better understanding, not only of ways of treating the disease, but also of the genetic profile of the population

As for polymorphism rs 16833974, statistically significant differences were found regarding the ethnicity variable, and the darker the skin color the greater the chance of having the polymorphism, *p*-value = 0.031.

## Financial support

This work was carried out with the financial support of Fundo de Apoio à Dermatologia (FUNADERM) of the Brazilian Society of Dermatology.

## Authors’ contributions

Amanda Hertz: Conception and planning of the study; obtaining, analysis, and interpretation of the data; critical review of the literature.

Luna Azulay-Abulafia: Effective participation in research orientation.

Adriana Paulino do Nascimento: Conception and planning of the study; obtaining, analysis, and interpretation of the data.

Cintya Yumi Ohara: Conception and planning of the study.

Fabio Chigres Kuschnir: Critical review of the literature; critical review of the manuscript.

Luís Cristovão Porto: Conception and planning of the study; obtaining, analysis, and interpretation of the data.

## Conflicts of interest

None declared.
